# Folic Acid–Fortified Iodized Salt and Serum Folate Levels in Reproductive-Aged Women of Rural India: A Nonrandomized Controlled Trial

**DOI:** 10.1001/jamanetworkopen.2024.1777

**Published:** 2024-03-08

**Authors:** Jogi V. Pattisapu, Vijayasekhar V. Manda, Madhu Narayana Rao Kottakki, Phani Madhavi Kajana, Vijaya Kancherla, Hayagriva Rao Bhaganagarapu, Vigneshwar Veerappan, Achuith Ediga, Venkatesh Mannar, Levente Diosady, Godfrey P. Oakley

**Affiliations:** 1 Pediatric Neurosurgery, University of Central Florida College of Medicine, Orlando; 2 Department of Neurosurgery, King George Hospital at Andhra Medical College, Visakhapatnam, India; 3 Department of Neurosurgery, Rangaraya Medical College, Kakinada, India; 4 Department of Community Medicine, Government Medical College, Rajamahendravaram, India; 5 Center for Spina Bifida Prevention, Department of Epidemiology, Rollins School of Public Health, Emory University, Atlanta, Georgia; 6 Institute of Health Sciences, University of Leeds, Leeds, United Kingdom; 7 Center for Global Engineering Myhal Centre, University of Toronto, Toronto, Ontario, Canada; 8 Department of Chemical Engineering and Applied Chemistry, University of Toronto, Toronto, Ontario, Canada

## Abstract

**Importance:**

India has a disproportionately high prevalence of neural tube defects, including spina bifida and anencephaly (SBA), causing a high number of stillbirths, elective pregnancy terminations, and child mortality; India contributes a large proportion of the global burden of SBA. Thirty years after folic acid was shown to be effective in reducing SBA prevalence, only about one-quarter of such births are prevented globally through cereal grain fortification.

**Objective:**

To determine the association of folic acid–fortified iodized salt with serum folate concentrations among nonpregnant and nonlactating women of reproductive age.

**Design, Setting, and Participants:**

This nonrandomized controlled trial using a preintervention and postintervention design was conducted in 4 rural villages in Southern India from July 1 to November 30, 2022. All households in the villages agreed to participate in the study. Preintervention and postintervention serum folate levels were analyzed among study participants at baseline and after 4 months, respectively.

**Intervention:**

Consumption of approximately 300 µg/d of folic acid using double fortified salt (folic acid plus iodine). Median serum folate concentrations were assessed at baseline and 4 months.

**Main Outcomes and Measures:**

Change in median serum folate levels between baseline and study end point as the primary outcome of the study.

**Results:**

A total of 83 nonpregnant nonlactating women aged 20 to 44 years (mean [SD] age, 30.9 [5.1] years) were eligible for the study and provided serum samples for analysis at baseline and the end point of the intervention. The median serum folate concentration increased from 14.6 (IQR, 11.2-20.6) nmol/L at baseline to 54.4 (IQR, 43.5-54.4) nmol/L at end of study, a 3.7-fold increase from baseline to study end point. Two-tailed Wilcoxon signed rank test showed the median difference in preintervention and postintervention serum folate concentrations to be highly significant (*P* < .001). The participants found the salt acceptable in color and taste.

**Conclusions and Relevance:**

Use of folic acid–fortified iodized salt was associated with increased serum folate concentrations in women of reproductive age. This novel evidence can inform public health policy to accelerate SBA prevention.

**Trial Registration:**

ClinicalTrials.gov Identifier: NCT06174883

## Key Points

**Question:**

Is folic acid–fortified iodized salt associated with increasing serum folate concentrations among nonpregnant and nonlactating women of reproductive age?

**Findings:**

This nonrandomized controlled trial of 83 female participants in rural India used a preintervention and postintervention design and found a statistically significant increase in the median serum folate concentration after 4 months of folic acid–fortified iodized salt consumption.

**Meaning:**

These findings suggest that folic acid–fortified iodized salt consumption may increase serum folate levels, which may in turn prevent folate deficiency leading to serious and fatal birth defects, especially spina bifida and anencephaly.

## Teaser

This nonrandomized controlled trial assesses serum folate concentrations after consumption of folic acid–fortified iodized salt by nonpregnant, nonlactating women aged 18 to 45 years in rural India.

## Introduction

Folic acid (vitamin B_9_) prevents severely disabling and life-threatening neural tube defects, such as spina bifida and anencephaly (SBA).^1^ Anencephaly is not compatible with life, and spina bifida can lead to a stillbirth or postpartum death, with significant health complications and permanent neurological disabilities in survivors.^2^ Approximately 260 000 SBA-affected births occur worldwide annually, with a global prevalence rate of 20 per 10 000 births.^3^ Limited surveillance studies in India suggest the prevalence is higher, at approximately 40 to 50 per 10 000 births, and contributes to a high number of stillbirths, elective pregnancy terminations, and deaths among neonates, infants, and children younger than 5 years.^4^

Spina bifida and anencephaly occur due to incomplete closure of the fetal neural fold during embryogenesis around day 28 after conception.^5^ Water-soluble folic acid is a crucial micronutrient required before and during early pregnancy for proper fetal DNA synthesis and complete neural tube closure.^5^ The US Preventive Services Task Force recommends all women planning to or who could become pregnant take a daily folic acid supplement containing 400 to 800 µg at least 1 month prior to anticipated conception and through the first 2 to 3 months of pregnancy.^6^ However, folic acid supplementation before pregnancy is a challenging public health intervention in many countries,^7^ and mandatory staple food fortification strategies have been more effective.^8,9,10,11,12^ About 60 countries are currently implementing mandatory fortification of wheat flour, maize flour, and/or rice with adequate levels of folic acid for prevention of SBA.^13^ In May 2023, the World Health Assembly passed a resolution recommending all member nations implement folic acid fortification to prevent SBA.^14^

The recent National Family Health Survey in India showed less than one-third of women consumed iron and folic acid supplements for at least 100 days during pregnancy,^15^ and even these women usually start supplement consumption too late during pregnancy to prevent SBA. In India and many countries, grain fortification with folic acid solves this limitation, and double-fortified salt with folic acid and iodine has the potential to accelerate the pace of SBA prevention.

Salt iodization has been a global success in preventing iodine deficiency; according to the Global Fortification Data Exchange, iodization was implemented sustainably in 126 countries through mandatory legislation and an additional 21 countries as a voluntary policy in the year 2022.^15^ Folic acid–fortified iodized salt can be an effective strategy to accelerate global SBA prevention.^16^ According to the 2018-2019 India Iodine Survey, 76% of Indian households have access to edible salt with adequate iodine (≥15 ppm).^17^ Use of iodized salt in India offers a promising opportunity for wide-scale SBA prevention.

To our knowledge, there are no published studies on the effect of double-fortified salt on serum folate levels among women of reproductive age. Our study aimed to assess serum folate concentrations after consumption of folic acid–fortified iodized salt (study salt) by nonpregnant, nonlactating women aged 18 to 45 years in rural India. These novel findings can support SBA prevention policies in India and other countries.

## Methods

### Study Setting and Design

This nonrandomized controlled trial used a preintervention and postintervention evaluation design to examine the outcomes of folic acid–fortified iodized salt in 4 rural villages in Southern India from July 1 to November 30, 2022 (total population of 1130, including 335 women of reproductive age [18-45 years]). This report follows the Transparent Reporting of Evaluations With Nonrandomized Designs (TREND) reporting guideline. The trial was registered retrospectively on December 8, 2023; the protocol is found in Supplement 1. The study team conducted multiple town hall meetings and seminars to educate the residents regarding SBA and the beneficial role of folic acid in preventing these birth defects. Women were invited to participate voluntarily after receiving information, and inclusion and exclusion criteria were applied to enroll participants. We included nonpregnant, nonlactating women who agreed to reside in their villages for the duration of the intervention and were willing to consume the study salt exclusively. We excluded women who were pregnant or lactating at the time of study recruitment, who were taking folic acid supplements, or who had health conditions such as malabsorption disorders, severe anemia (hemoglobin level <8.0 g/dL [to convert to g/L, multiply by 10.0]), uncontrolled hypertension (systolic blood pressure ≥140 mm Hg or diastolic blood pressure ≥90 mm Hg), HIV infection, cancer, active tuberculosis, or malaria infection. Additionally, we excluded women with a prior pregnancy affected by SBA or taking regular medications for preexisting medical conditions.

Signed written consent was obtained from all participants in the local language (Telugu) prior to initiating the study. The study was approved by the Institutional Review Board Ethics Committees of Andhra Medical College, Visakhapatnam, India, and Emory University, Atlanta, Georgia. Approval from the District Collector’s office was obtained, which facilitated cooperation from the villagers, their leaders, and local administrative officers. The medical team performed routine assessments of the villagers during scheduled visits and monitored for any new health complaints or adverse events. A local study coordinator was recruited for each village to supervise the implementation and serve as a liaison between the participants and study team. A study center was established in a central location in each of the 4 villages, and the collaborator with trained data collectors ensured that the study methodology was uniformly maintained. Most of the data collection occurred at these centrally located study centers on a specified date; a small proportion of participants who were not able to participate at the appointed time was recruited through door-to-door surveys or blood sampling as needed.

Paper surveys used to screen study participants collected basic demographic information, use of folic acid supplements, and health-related variables. Daily dietary folate intake was not assessed in our study, and periodic interviews assessed whether participants consumed folic acid supplements.

### Preparation of Folic Acid–Fortified Iodized Salt

The study salt was custom made by Wella Nutrologicals by mixing commercially available iodized table salt (Tata) with an alkaline folic acid solution in a batch ribbon blender. The folic acid formulation has limited solubility at a pH of less than 8 and thus was solubilized by buffering to a pH of greater than 9. Sodium carbonate (21.2 g) was dissolved in 900 mL of treated water and allowed to cool, after which 10 g of folic acid was added to this solution and stirred to dissolve completely. The solution was topped to a volume of 1 L with additional water, and 4 L of the solution (containing 1% weight to volume ratio of folic acid) was sprayed evenly on 1 ton of iodized salt in a batch ribbon blender and mixed for 10 minutes. The study salt was tested, labeled, and packed in 1-kg double-polylaminated plastic bags. Laboratory testing confirmed stable iodine and folic acid levels in the test salt mixture for 8 months of storage at ambient temperatures in the field.

### Standardizing Salt Consumption Across Participants

Random salt consumption measurements by weight were conducted prior to distributing the study salt in the participating villages. The average family ingested approximately 8.75 g/person, which agrees with established average daily salt intake in India.^18^ Therefore, we estimated that participants in our study consumed about 300 µg/d of folic acid using the study salt (containing 350 µg of folic acid per 10 g of iodized salt). In a voluntary exchange program, the currently used home stock was replaced with the study salt in labeled containers and provided to all households in the village free of cost to avoid access to non—folic acid–fortified commercial salt. The village grocers also cooperated by not selling commercial salt until the study ended. The study team members maintained routine communications with the villagers and local health care professionals to ensure adherence and monitoring of any potential adverse effects. Frequent reminders helped avoidance of folic acid supplements or consumption of nonstudy salt. The local medical nodal officer was engaged during the study period, and any questions or concerns were immediately addressed.

Strict security protocols were used during data collection and storage to ensure confidentiality. The paper surveys and laboratory reports were securely transported using stringent protocols to a locked central data storage facility at the Dr Pattisapu Ramajogi Gangadharam Academic and Research Cell in the Department of Neurosurgery, Andhra Medical College.

### Serum Folate Concentration Assessment

Serum folate analyses were performed on nonfasting blood samples at baseline and at the end point. Specimens were transported on ice immediately to the laboratory (Quantum Diagnostics). Phlebotomists collecting the blood were not blinded to the intervention. However, the samples were subsequently coded such that the laboratory technicians analyzing the samples were blinded to their origin. A folate assay (ARCHITECT [Abbott Laboratories]) was used to analyze serum folate concentrations. Analytic performance of this method has been validated.^19^ The test involves a 2-step, chemiluminescent microparticle folate binding protein assay for quantitative determination in human serum, plasma, and red blood cells on the ARCHITECT i-System. In our samples, the test performance allowed capture of the maximum value of serum folate level up to 54.4 nmol/L (to convert to ng/mL, divide by 2.266).

### Statistical Analysis

A 2-fold increase in the mean serum folate level at study power set at 80% required a sample size of 10 participants (2-sided 1-sample *t* test for mean). Our analytical sample of 83 participants provided a study power of greater than 99%. Key descriptive statistics including median, IQR, and range were estimated for baseline and end point serum folate test results. Analysis was restricted to 83 of the 89 participants as they provided blood samples at both preintervention and postintervention points. As the folate assay measured serum folate only to the concentration of 54.4 nmol/L, we used the median as the measure of central tendency. Statistical differences in the median comparing preintervention and postintervention serum folate concentrations were assessed using 2-tailed Wilcoxon signed rank test for paired samples. We hypothesized a significant difference in the median serum folate concentrations from baseline to the end point of the study, with a 2-sided *P* <.05 indicating statistical significance. All data were analyzed using SAS, version 9.4 (SAS Institute Inc).

## Results

Of the 89 women who were eligible and enrolled in the study at baseline (July 2022), 83 provided blood samples at the baseline and at the postintervention end point (November 2022) for serum folate analysis and were included in our analytic sample. The mean (SD) age of women in our analytic sample was 30.9 (5.1) years (median age, 30 years; range, 20-44 years). The median serum folate concentration was 14.6 (IQR, 11.2-20.6) nmol/L at baseline and 54.4 (IQR, 43.5-54.4) nmol/L at 4 months, a 3.7-fold increase from baseline to study end point. The results of a 2-tailed Wilcoxon signed-rank test indicated that this was a significant difference (*P* < .001).

**Figure 1.**
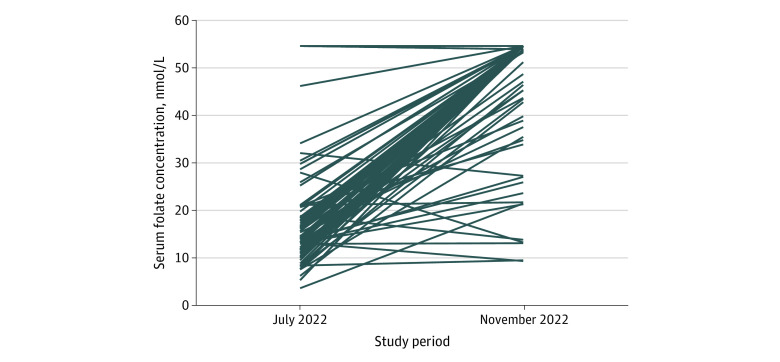
Paired Profiles for Serum Folate Concentrations Before and After Folic Acid–Fortified Iodized Salt Intervention The laboratory folate assay detected serum folate concentration only to 54.4 nmol/L (to convert to ng/mL, divide by 2.266), leading to a ceiling effect.

Figure 1 shows individual-level change in serum folate concentrations from baseline to study end point. More than 90% of the participants experienced an increase in their serum folate level at the end of the intervention. One participant had the same folate concentration at baseline and end point (54.4 nmol/L). Six participants had a decrease in their serum folate concentrations between baseline and postintervention assessments; of these, only 2 had baseline serum folate levels that were less than 20 nmol/L, and their postintervention levels also remained below 20 nmol/L. No noticeable clinical profile emerged indicating folate deficiency in participants whose serum folate concentrations did not increase after intervention.

**Figure 2.**
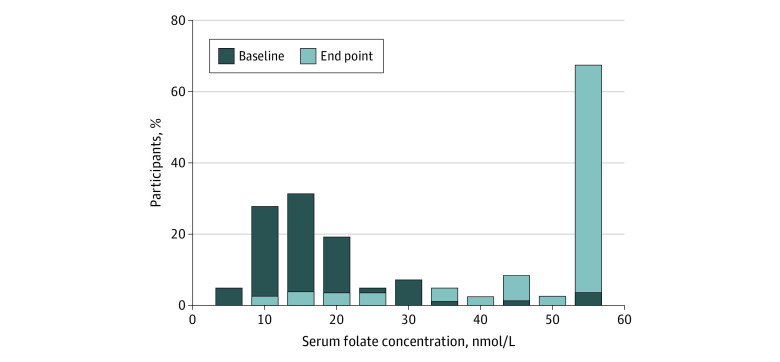
Distribution of Serum Folate Concentrations During Preintervention and Postintervention Evaluations The laboratory folate assay detected serum folate concentration only to 54.4 nmol/L (to convert to ng/mL, divide by 2.266), leading to a ceiling effect.

Figure 2 represents the shift in the distribution of serum folate concentrations from baseline to study end point. As mentioned earlier, the ARCHITECT assay measured serum folate levels up to 54.4 nmol/L. There was a remarkable shift in the distribution of serum folate levels to the right in postintervention samples, indicating an improvement in the main outcome measure of the study. We noted that only 3 participants (3.6%) had reached the test ceiling (54.5 nmol/L) at baseline; however, this proportion increased to 48 (57.8%) at the end of the intervention.

The study salt was acceptable in its appearance and taste as reported during town hall meetings and door-to-door interviews. Four minor health complaints, including headache (1 participant), fever (2 participants), and stomach pain (1 participant), were deemed unrelated to the study salt consumption by the local medical nodal officer. The study salt was stable for 8 months at room temperature in a community setting, retaining its iodine and folic acid levels on repeated testing.

## Discussion

Although robust surveillance data are lacking regarding SBA prevalence in India, it is estimated that the subcontinent accounts for nearly one-third of the global burden of SBA, contributing to a high number of elective terminations of pregnancies, stillbirths, and deaths among children younger than 5 years.^3^ In our study, we found a 3.7-fold increase in median serum folate levels among participants after they consumed approximately 300 µg/d of folic acid provided by the study salt. This finding suggests that folic acid–fortified iodized salt may improve folate concentrations in women of reproductive age, offering another opportunity for prevention of SBA in India and other countries. Given the high use of iodized salt in Indian households, this option offers a viable fortification strategy to deliver the recommended daily dose of folic acid equitably on a national scale. The folic acid concentration can be adjusted for lower daily salt consumption, keeping in focus the goal to reduce sodium intake. The high potential of accelerating the global prevention of folic acid–preventable SBA through use of folic acid–fortified iodized salt was modeled recently.^16^ Our results provide the needed evidence to show the effectiveness of the intervention to inform SBA prevention policy. As India and other countries are working toward achieving 2030 Sustainable Development Goals on preventable mortality among neonates and children younger than 5 years,^20^ folic acid fortification of iodized salt can serve as a promising strategy.

In our study, we found that after 4 months of daily consumption of approximately 300 µg of folic acid from fortified iodized salt, the median serum folate concentration of study participants increased from 14.6 to 54.4 nmol/L (a 3.7-fold increase). Another 3-month study in women of reproductive age consuming 100-µg and 400-µg folic acid supplement pills daily^21^ reported serum folate level increases by 2.0-fold and 3.8-fold, respectively.

We also found that individual serum folate levels did not increase in 7 of the 83 participants. Although we cannot confidently explain the reason for this finding, we speculate that it may be due to poor adherence or other clinical considerations that were beyond the scope of our study.

Data are available about the feasibility of double fortification of salt with iodine and folic acid that remains stable during storage.^22^ Iodine and folic acid content in our study salt samples was found to be stable at 8 months after production while stored at room temperature in the community setting. To our knowledge, large-scale production of folic acid–fortified iodized salt has not yet been implemented; our study findings provide support for such an undertaking.

There is an established association between achieving optimal maternal serum folate levels and SBA prevention in offspring. In 1995, Daly et al^23^ showed a strong inverse dose-response relationship between maternal plasma or red blood cell folate concentration and SBA prevalence. Our study found an association between daily consumption of approximately 300 µg in folic acid–fortified iodized salt and increased serum folate concentration.

Double fortification of salt with iodine and folic acid could overcome current challenges with dietary variances and low compliance of supplement intake in India. It can provide women of childbearing age the recommended level of folic acid before and during early pregnancy, a critical intervention window for prevention of SBA. Using folic acid–fortified iodized salt provides a global opportunity to prevent thousands of SBA cases; achieve the World Health Organization’s Sustainable Development Goal 3, which is aimed at reducing neonatal morality and deaths among children younger than 5 years^20^; and provide equitable health and well-being for all.

### Strengths and Limitations

The key strength of our study is that the selected villages were remote and had limited external influences on the intervention. There was high adherence, as the study salt was well accepted and all villagers consumed it, and there was high participant retention. The participants were monitored periodically, and there were no competing interventions or events that could have affected the study outcomes. The salt was stable at community storage settings.

This study also has some limitations. Since our study was limited to 4 villages (83 participants), the findings are not representative of the general population in India. Again, we elected to use the recommended dose of folic acid to assess the vehicle (salt) and recognize the option to adjust the concentration according to local needs and variances. Another limitation is that we did not conduct a comparative analysis of folic acid–fortified salt with a control (non–folic acid consumption) group or with participants receiving other food vehicles.

## Conclusions

This nonrandomized controlled trial provides novel evidence that folic acid–fortified iodized salt consumption increases serum folate levels among women of reproductive age with the potential to prevent SBA in their offspring. This finding offers an opportunity for public health policy makers in India and other countries to consider effective folic acid fortification programs.

## Notes

Supplement 1.
Trial Protocol

Supplement 2.
Data Sharing Statement
