# Supplementary material for: Folic Acid–Fortified Iodized Salt and Serum Folate Levels in Reproductive-Aged Women of Rural India: A Nonrandomized Controlled Trial
Source: JAMA Netw Open. 2024 Mar 8;7(3):e241777. doi: 10.1001/jamanetworkopen.2024.1777 (PMC10924245; doi:10.1001/jamanetworkopen.2024.1777)
Supplement: Supplement 1. — Trial Protocol [file jamanetwopen-e241777-s001.pdf]

TITLE:

EFFECTIVENESS OF FOLIC ACID FORTIFIED SALT IN INCREASING THE BLOOD FOLATE LEVELS AMONG WOMEN OF REPRODUCTIVE AGE TO PREVENT NEURAL TUBE DEFECTS – PRE- AND POST-INTERVENTION STUDY.

Study Collaborators:

1. Dr. M. V. Vijaya Sekhar, Professor & HOD, Dept of Neurosurgery, RMC Kakinada

2. Dr. B. Hayagreeva Rao, Professor, Dept of Neurosurgery, AMC, Visakhapatnam

3. Dr. T. Phaneeswar, Assistant Professor, Dept of Neurosurgery, AMC, Visakhapatnam

4. Dr. K.V. Phani Madhavi, Assistant Professor, Dept of Community Medicine, AMC, Visakhapatnam.

5. Dr. E. Achuith, Post-graduate, Dept of Neurosurgery, AMC, Visakhapatnam.

6. Dr.Godfrey Oakley, Dept of Epidemiology, Rollins School of Public Health Emory University, Atlanta, GA, USA
7. Dr.Vijaya Kancherla, Dept of Epidemiology, Rollins School of Public Health Emory University, Atlanta, GA, USA
8. Dr. Jogi Pattisapu, Paediatric Neurosurgery, Orlando FL, USA

CONSULTANTS – Prof. Venkatesh Mannar, University of Toronto, Canada
Prof. LeventeDiosady, University of Toronto, Canada

INTRODUCTION:

FOLIC ACID is a water soluble naturally occurring vitamin needed by pregnant women before and after pregnancy. Deficiency of folate can increase the risk of neural tube defects (NTD) in the baby. There are over 260,000 cases of NTDs each year globally, ranging from 1 to 11 per 1000 births.[1,2]

The incidence of NTD is significantly higher in India (7.48 per 1000 births) as compared to developed countries like England, Germany , US (where it is less than 1 per 1000 live births)[3]The incidence of NTD in Andhra Pradesh was found to be 5.08 in a study done by Rai SK et al in 2016.[3]

Fortunately, research has shown that folic acid taken by pregnant women before and during pregnancy can prevent NTD. consuming folic acid as vitamin supplementation or through folic acid fortified foods (wheat and maize flour) is a cost effective, safe, and provides significant protection against NTD. This protective effect has been consistently proven in various studies since early 1900s. In 1998 it became mandatory in the United States to fortify grain products with folic acid which led to a 26% reduction in neural tube birth defects (WHO, 2006).[4]

As of year 2017, almost 60 countries have a mandatory policy to adequately fortify their wheat and/or maize flour with folic acid, achieving about 20% prevention of neural tube defects prevention worldwide (Kancherla et al., 2018).[5]

Unfortunately, flour and its derivatives centrally/commercially processed derivatives are not universally consumed and a large segment of the population is not benefiting.

Salt is an ideal vehicle for fortification because its consumption is universal and stable throughout the seasons irrespective of economic status, it is centrally processed with established distribution channels, and is generally purchased as opposed to bartered (WHO, 2008).[6]

Now over 70% of households in developing countries have access to iodized salt (Mannar, 2007).[7]

Considering the success of salt iodization, it is logical to use the existing salt iodization infrastructure to also provide folic acid through salt. In India, salt is one of the most widely consumed fortifiable staples. Recent technological advances have informed the development of a low-cost formulation of folic acid-fortified salt (QFS) - i.e., iron, iodine, folic acid, and vitamin B12 (Diosady, Mannar).[8]

This QFS is formulated to prevent chemical interactions of micronutrients and maintain stability in tropical conditions, and is ready for use in randomized efficacy trials in human populations.

Folic acid can be added to iodized salt. Several studies have demonstrated the stability of folic acid in salt during storage (Li et al., 2011[9]; Sangakkara, 2011.,[10]McGee et al., 2017;[11]Vinodkumar& Rajagopalan, 2009)[12], after cooking (Vinodkumar& Rajagopalan, 2009). Studies of color changes to salt after fortification with folic acid have reported mixed results, either no changes (Li et al., 2011; Vinodkumar& Rajagopalan, 2009) or yellowing (McGee et al., 2017; Sangakkara, 2011). Although salt fortification with iodine and folic acid is technically feasible and has been demonstrated in the lab (Li et al., 2011), experiences in large-scale

production are underway, and effectiveness on improving blood folate concentrations among target populations are needed.

Salt is an ideal vehicle for fortification because its consumption is universal and stable throughout the seasons irrespective of economic status, it is centrally processed with established distribution channels. Now over 70% of households in developing countries have access to iodized salt (Mannar, 2007). Considering the success of salt iodization, it is logical to use the existing salt iodization infrastructure to also provide folic acid through salt.

A large study in China has shown periodic improvements in both serum/plasma andRBC folate levels after folic acid supplement intake among women of reproductive age at months 1, 3 and 6 and 3 months after discontinuation of supplementation.[13]

Sharp increases in both serum/plasma and RBC folate levels were observed after one month of folic acid intake, and additional increases were noted at months 3 and 6 for RBC folate concentration among study participants, at a dose of 400 mcg/day of folic acid intake.[13]

Rationale for the Proposed Study:

As on date, there are no studies that have examined the relationship between consuming folic acid fortified iodised salt and serum folate levels in south India. Hence this study is being done to get an insight into impact of folic acid fortified salt on serum folate levels.

AIM:

To assess increase in blood folate concentrations among women of reproductive age after they consume folic acid-fortified iodized salt.

OBJECTIVES :

1. To examine the effect of consumption of salt fortified with folic acid on serum/plasma folate concentrations among women of reproductive age.
2. To estimate the raise in serum iron, B12 and folate levels at various study intervalsi.e baseline, at the end of 1month and 3 months.

Study setting: The study will be carried out in the selected tribal villages ofKoyyuru Mandal,Alluri Sitaramaraju District with apopulation ofTummalabanda, Borrampeta,

Ankampalem, Nelipudi, Balaram, Kantaram and Bakuluru

Hukumpeta Mandal, Alluri SitaramarajuDist
Gattum, Velagapadu, Durgam, Galipadu, Karakavalasa, Dalemputtu, Jangamputtu, Maddiputtu, Kimuduputtu, Cheekatiputtu, Juvvipadu, Jeelugulaputtu, Billaputtu and A. Gattum

Study duration: 3 months

Study Design:

An experimental, pre-post intervention study, comparing change in serum/plasma folate concentrations in study participants measured at baseline (pre-intervention) and at the end of selected time points (1 month and 3 months) post-intervention.

Sample size calculation:

120 women of reproductive age will be given fortified salt (60 women Double Fortified Salt and 60 Women Quadrable Fortified Salt) 60 women residing inTummalabanda, Borrampeta, Ankampalem, Nelipudi, Balaram, Kantaram and Bakuluruwill be given Double Fortified Salt and 60 women residing in Gattum, Velagapadu, Durgam, Galipadu, Karakavalasa, Dalemputtu, Jangamputtu, Maddiputtu, Kimuduputtu, Cheekatiputtu, Juvvipadu, Jeelugulaputtu, Billaputtu and A. Gattum will be given Quadrable Fortified Salt.

tribal villages will be recruited from the community to participate in the study.

Intervention model description:

1. One arm: Quadruple-fortified salt (QFS; i.e., iron, iodine, folic acid, and vitamin B12)
2. Other arm: Double-fortified salt (DFS; i.e., Folic Acid and iodine)

- **One arm: We** will recruit 60 non-pregnant WRA residing in the catchment area of 1-2 primary health care units (PHCUs) from the ……………. of …………………... Recruited women's households will receive Quadruple-fortified salt every 4 weeks.
- **Other arm:**We will recruit 60 non-pregnant WRA residing in the catchment area of 1-2 primary health care units (PHCUs) from the ……………..of …………………... Recruited women's households will receive Double-fortified salt every 4 weeks.

The FA-salt will be formulated locally to provide the participant, and their family, with 400 mcg of FA for 10 grams of iodized salt in daily diet. We will not modify the nationally recommended amount of iodine content.

Subject Selection Criteria:

Inclusion Criteria:

- Willing to participate in the study and who give consent.
- Participant should be capable of comprehending and complying with study requirements (to only use the given fortified salt during the study period).
- Participants should be Married Women between 18 and 49 years of age.

- Sexually active females of child bearing potential willing to conceive and continue pregnancy.
- Participants should be residing in the study area for the entire duration of the study.

Exclusion Criteria:

- Females who do not have childbearing potential (if they have undergone bilateral tubal ligation or occlusion, or hysterectomy, or bilateral ovariectomy are post-menopausal)
- Pregnant and Lactating at the time of recruitment
- **Known to be suffering with Malabsorption disorders (medical condition that may affect vitamin B12 absorption or metabolism)**
- Severe anaemia (Haemoglobin <8.0 g/dL)
- HIV, active tuberculosis disease, or malaria infection
- Severe hypertension (SBP$\geq$140 mm Hg or DBP$\geq$90 mm Hg)
- Other serious pre-existing medical condition (i.e., defined as the need for regular medication use)
- **Participants taking multivitamins or folic acid supplements.**
- Participants with a history of delivering an offspring with NTD in the past will be excluded as these women are considered to be at a high risk of recurrence, and require higher dose of folic acid than what is available in fortified foods.
- On ethical grounds, women with a history of delivering an offspring with NTD will receive a 6-month supply of 5 mg/day of folic acid supplement pills from the study team, but will not be recruited into the study.
- **Pregnancy**. Women who become pregnant during the study will receive folic acid supplement pills (400 mcg/day) and will be analyzed as a sub-group.
- Intramuscular or intravenous interventions containing vitamin B12 or iron in the past 3 months.

- Any other condition that in the opinion of the investigator would jeopardize the safety or rights of the volunteer participating n the study or make it unlikely that the participant could follow the protocol.

Intervention Model Description: Folic acid-Fortified Salt (QFS)

Salt fortified with iron, iodine, folic acid, and vitamin B12 will be given free of cost to families of the participants for daily consumption for a period of 3 months. The average household consumption of salt in Andhra Pradesh is 10.41 grams as suggested by a study done by Johnson C et al in 2017.

The FA-salt will provide the participant, and their family, with 400 mcg of FA for 10 grams of iodized salt in daily diet.

As folic acid is a water-soluble vitamin, over consumption of fortified salt will not cause any harm. Consumption of folic acid as levels as high as 1000mcg /day has no side effect and is well tolerated without any side effects as suggested by institute of medicine, food and nutrition board.

Investigational Product:

Ingredients of the Investigation product :

Folic acid-Fortified Salt (QFS)

Iron (….. % per KG)

Iodine (….. % per KG)

Folic acid (….. % per KG)

Vitamin B12 (….. % per KG)

Folic acid-Fortified Salt (DFS)

Iodine (….. % per KG)

Folic Acid(….. % per KG)

Data Collection:

Data will be collected by trained health extension workers (HEWs) after receiving written informed consent from participants and their family members at three time points (baseline, and post-intervention months 1,3). All data collection will be done at residence of participants. Strict personal protection protocols will be followed by the study staff and households during the interview to ensure social distancing and safe draws of blood using face masks to address the COVID-19 pandemic guidelines.

Trained phlebotomists will accompany HEWs during household visits to draw blood for the required blood tests. HEWs will implement short questionnaires at baseline and the two follow-up visits. Questionnaires will be paper based and will be filled concurrently by HEWs as they interview participants in local language.

Subject matter experts in the study team will develop the baseline and follow-up questionnaires. Survey protocol will be developed by the study team to guarantee preservation, retention and retrieval of information and allow easy access for verification, audit and inspection.

The baseline questionnaire will include information on demographics, (household size, income, age) medical history, and use of supplements, and other factors relevant to the study. Follow-up questionnaires will assess adherence to daily intake of folic acid fortified salt, any initiation of folic acid supplements since the last survey, and other relevant variables.

Blood sample collection will occur during the baseline survey and two subsequent follow-up household visits. Blood will be transported to the lab following protocol to be stored and analysed for serum/folate concentrations. Serum folate analysis will be conducted at the standard lab for baseline, 1st and 3rd month samples. All measures will be taken to maintain cold chain and standard laboratory measures for serum folate analysis. Standard testing procedures recommended will be adhered to while analysing blood samples.

Laboratory Analysis: Serum folate samples will be analysed at two time periods, including baseline (pre-intervention) and at the end 1st month and 3rd month post intervention. The

serum/plasma folate analysis will be conducted at the standard lab, using a commercial protein binding assay.

Baseline parameters (0 visit)

- CBC
- Thyroid Profile
- HIV
- HBS AG
- UPT
- Stool examination to exclude malabsorption
- Serum calcium
- serum folate concentrations, nm
- Haemoglobin concentrations, g/dL
- Vitamin B12 concentrations, pmol/L

At the end of 1 month (1st visit):

- Serum folate concentrations, nm
- Haemoglobin concentrations, g/dL
- Vitamin B12 concentrations, pmol/L

At the end of 3 months (2nd visit):

- Serum folate concentrations, nm
- Haemoglobin concentrations, g/dL
- Vitamin B12 concentrations, pmol/L

Monitoring of salt intake: check for compliance by making house- house visits and checking their kitchen and motivate/ counsel them to use salt daily with the help of health team (workers/field staff). The above biochemical parameters are done for all women in reproductive age group. Apart from study participants the above biochemical tests will be done on 1 or 2 adults in each house to ensure whether the family members are consuming salt every day. Rise in the above biochemical parameters ensures that there is regular intake.

Low level of above biochemical parameters could be noticed IN 2 CONDITIONS:

1. If salt is not consumed regularly by the family.
2. Presence of genetic conditions which may cause malabsorption of folic acid in which case further Evaluation of the genetic conditions will be done with the help of MDRU, KGH, Visakhapatnam.

In case if there are no genetic defects noticed, those families will be again counselled for regular intake of salt. HH visits will be made by the ground level field staff to motivate the families to improve their compliance.

Data Analysis:

Summary statistics for demographic, maternal health and other characteristics will be presented for baseline and follow-up data. Data will be assessed for normality and homogeneity of variance. The serum/plasma will be summarized using geometric means and their standard deviations. If the measures of central tendency are skewed (based on Shapiro-Wilk test), data will be log-transformed for analysis. The student’s paired t-test will be used to compare two time points. When data are not normally distributed, Wilcoxon signed-rank tests will be performed. Statistical analyses will be performed using SPSS 22.0 software. P values at or less than 0.05 will be considered statistically significant.

Ethical Considerations

Study Approvals: Approval of study protocol and all associated data collection surveys and consent forms will be sought from the Institutional Ethics Review Committee, AMC, KGH, Visakhapatnam. Study permissions will also be sought from the local health authorities PO, ITDA of tribal areas for conducting the study.

Informed Consent:

Study participants will be introduced to the study before initiation of enrolment by trained HEW. Informed oral and written consent will be obtained for the baseline as well as for two follow-up home visits. Written consent forms will be developed by trained translators in the local language, and participants will retain a copy of such letter. The choice to participate will be completely voluntary and every participant, even after giving consent to participate, shall retain the right to opt out of the study at any time. All information obtained shall strictly be kept confidential and shall only be used for purposes of the said study. All data will be stored in a secured area at Department of Neurosurgery, AMC and only study personnel will have access to these data. All data will be analysed by study analysts on a secure server, and any data sharing between co-investigators will be de-identified.

Study Result Dissemination

Study results will be determined and reports written. The report will be delivered to the concerned health care authorities, stating whether or not the data permit the rejection of the hypothesis that ‘consuming folic acid fortified iodized salt does not increase serum/ plasma concentrations in the target population of women of reproductive age. We will draft periodic reports starting at one month of postintervention serum/plasma folate samples comparing to the baseline levels. Thus, reports will be prepared in a timely manner after baseline, 1 month and 3 months of serum/plasma folate analyses. All the reports will be disseminated to relevant stakeholders through virtual web meetings (adhering to COVID-19 pandemic safety protocols) and through publication of findings in peer-reviewed scientific journals. The appropriate health care authorities / local PO, ITDA and others will also be informed of the study protocol and study results.

Expected Study Outcome

The proposed study will provide new evidence of whether receiving 400 mcg/day of folic acid by consumption of 10 grams of folic acid fortified salt would lead to increased serum/plasma folate concentrations among women of reproductive age. We hope these timely results will prompt regulations that lead quickly to mandatory folic acid fortification of iodized salt.

The results of our study could also serve as a pilot to inform additional studies to collect further evidence about the effects of consuming folic acid fortified salt. By showing that even as little as one month's consumption of folic acid fortified salt triple serum/plasma folate concentrations known to prevent NTDs, our study can provide required evidence and stimulus to help to mobilize a national program for folic acid fortification of iodized salt in India as well as other countries. Additionally, upon implementation of national salt fortification programs with folic acid, we can expect significant reductions in folate deficiency anemia in the population (Odewole et al., 2013), and reductions in child mortality associated to preventable NTDs (Kancherla et al., 2019).

REFERRENCES:

1. Verma IC, Mathews AR. Congenital malformations in India. In: Satyavati GV, editor. Peoples of India: Some Genetical Aspects. New Delhi: Indian Council of Medical Research; 1983. p. 70.

2.Kumari BS, Panda R, Rath S. Neural tube defect: Epidemiologic and demographic implication. IOSR J Dent Med Sci 2014;13:1-4.

2.Kumari BS, Panda R, Rath S. Neural tube defect: Epidemiologic and demographic implication. IOSR J Dent Med Sci 2014;13:1-4.

3.Rai SK, Singh R, Pandey S, Singh K, Shinde N, Rai S, et al. High incidence of neural tube defects in Northern part of India. Asian J Neurosurg2016;11:352-5.

4. WHO. (2006). *Guidelines on food fortification with micronutrients.* (L. Allen, B. d. Benoist, O. Dary, & R. Hurrell, Eds.) Retrieved March 7, 2012, from World Health Organization: http://whqlibdoc.who.int/publications/2006/9241594012.

5. Kancherla, V., Wagh, K., Johnson, Q., Oakley, G.P. (2018). A 2017 global update on folic acid-preventable spina bifida and anencephaly. *Birth Defects Res*. 110(14):1139-1147.

6. WHO. (2008). *Salt as a Vehicle for Fortification.* Luxembourg: World Health Organization Expert Consultation. Retrieved August 5, 2014, from http://whqlibdoc.who.int/publications/2008/9789241596787_eng.pdf.

7. Mannar, M. (2007). Eliminating iodine deficiency: learning from an untold public health success story. (K. Engesveen, Ed.) *SCN News (United Nations System Standing Committee on Nutrition)*(35), pp. 3-4. Retrieved August 5, 2014, from United Nations Standing Committee on Nutrition: http://www.unsystem.org/scn/Publications/SCNNews/scnnews35.pdf.

8. Diosady, L.L., Mannar, M.G.V., Krishnaswamy, K., 2019. Improving the lives of millions through new double fortification of salt technology. Matern. Child Nutr. 15 https://doi.org/10.1111/mcn.12773 e12773, 2019/05/01.

9. Li, Y. O., Diosady, L. L., & Wesley, A. S. (2011). Folic acid fortification through existing fortified foods: iodized salt and vitamin A-fortified sugar. *Food and Nutrition Bulletin*, **32**(1), 35–41.

10. Sangakkara, A. R. (2011). *Double fortification of salt with folic acid and iodine*. Retrieved from https://tspace-library-utoronto-ca.proxy.library.emory.edu/bitstream/1807/31430/1/Sangakkara_Angjalie_R_201111_MASc_thesis.pdf

11. McGee, E. J. T., Sangakkara, A. R., &Diosady, L. L. (2017). Double fortification of salt with folic acid and iodine. *Journal of Food Engineering*, **198**, 72–80.

12. Vinodkumar, M., & Rajagopalan, S. (2009). Multiple micronutrient fortification of salt. *European Journal of Clinical Nutrition*, **63**, 437–445.

13. Crider, K. S, Zhu, J. H, Hao, L., et al. (2011). MTHFR 677C->T genotype is associated with folate and homocysteine concentrations in a large, population-based, double-blind trial of folic acid supplementation. *Am J Clin Nutr*, **93**(6), 1365-1372.

Questionnaire

1. **Name of the head of the family**
2. **Age:**
3. **Family size**
4. **Income**
5. **Education of the participant**

Study variables: basic socio-demographic data,

H/O consanguity

Alcohol

Radiation exposure

Usage of Sod valproate /Carbamazepine

Chromosomal abnormalities (trisomy of 13,15)

Maternal malnutrition

Diagnostics:

BASELINE SERUM FOLIC ACID LEVELS

SERUM IRON LEVELS.

Hb %

HIV

Permissions:

Institutional Ethics Committee, AMC, KGH approval will be obtained before conducting the study.

Informed written consent will be obtained from the individuals of the families.

Confidentiality of the study participants will be maintained.

Outcome Measures

Primary Outcome Measures :

1. Concentrations of serum folate [Time Frame: End of 3months]

   serum folate concentrations, nmol/dL

2. Concentrations of haemoglobin [Time Frame: End of 3 months]

Haemoglobin concentrations, g/dL

3. Concentrations of vitamin B12 [Time Frame: End of 3 months]

Vitamin B12 concentrations, pmol/L

INFORMATION SHEET

I ----------- doing a study titled - **EFFECTIVENESS OF FOLIC ACID FORTIFIED SALT IN INCREASEING THE BLOOD FOLATE LEVELS AMONG WOMEN OF REPRODUCTIVE AGE – AN INTERVENTIONAL STUDY**

Before participating in this study, you should know the following details.

1.You will be given an informed consent form and your written consent is mandatory to participate in this study.

2. Age, Gender, Occupation, Education, Area of residence, marital status, personnel and past medical history will be collected and noted.

3. Vital data /General/Systemic examination of the patient will be noted down.

4. Investigator will collect 10ml of blood to do all blood investigations

4) It will take 40 to 50 minutes to take history, physical examination and sample collection

5) your decision to participate in the study is completely Voluntary.

6) During the period of study if any complications occur the investigator with the help of KGH Neurosurgery department specialists will treat the patient and take responsibility for the patient.

7) By participating in this study, you will help the community as the information provided here and the result of laboratory tests done will be analysed scientifically and help us in understanding and treating neural tube defects. Thank you for your time.

Signature of the Patient/LAR

Signature of Investigator

□□□□□□□□□□□□

----------------- అనే నేను

“పునరుత్పత్తివయస్సుగలస్త్రీలలోరక్తపుఫోలేట్స్థాయిలనుపెంచడంలో ఫోలిక్ యాసిడ్ కలిపిన సాల్ట్ (□□□□□□□□□)యొక్కసమర్థత“అనేఒకఅధ్యయనంచేస్తున్నాను

ఈఅధ్యయనంలోపాల్గొనేముందు, మీరుఈక్రిందివివరాలనుతెలుసుకోవాలి.

1.మీకుసమాచారసమ్మతిపత్రంఇవ్వబడుతుందిమరియుఈఅధ్యయనంలోపాల్గొనడానికిమీవ్రాతపూర్వకసమ్మతితప్పనిసరి.

2. వయస్సు, లింగం, వృత్తి, విద్య, నివాసప్రాంతం, వైవాహికస్థితి,మరియుగతవైద్యచరిత్రసేకరించబడుతుంది

3. మీఎత్తు, బరువు, రక్తపోటుకొలతనమోదు□□□□□□□□□□

4. అన్నిరక్తపరిశోధనలుచేయడానికిపరిశోధకుడు 10ml రక్తాన్నిసేకరిస్తారు

4) శారీరకపరీక్షమరియునమూనాసేకరణనుతీసుకోవడానికి 40 నుండి 50 నిమిషాలుపడుతుంది

5) అధ్యయనంలోపాల్గొనాలనేమీనిర్ణయంపూర్తిగాస్వచ్ఛందమైనది.

6) అధ్యయనకాలంలోఏవైనాసమస్యలుఎదురైతే, KGH □□□□□□□□□□□□□□విభాగంనిపుణులసహాయంతోపరిశోధకుడురోగికిచికిత్సచేస్తారుమరియురోగికిబాధ్యతవహిస్తారు.

7) ఈఅధ్యయనంలోపాల్గొనడంద్వారా, ఇక్కడఅందించినసమాచారంమరియుచేసినప్రయోగశాలపరీక్షలఫలితాలుశాస్త్రీయంగావిశ్లేషించబడినందునమీరుసంఘానికిసహాయంచేస్తారుమరియున్యూరల్ట్యూబ్లోపాలనుఅర్థంచేసుకోవడంలోమరియుచికిత్సచేయడంలోమాకుసహాయంచేస్తారు. నీసమయానికిధన్యవాదాలు.

□□□□□□□□□□□□□□□□□□□□□□□□□□□□□

తేద్తేద్

Informed Consent Form

Study title: EFFECTIVENESS OF FOLIC ACID FORTIFIED SALT IN INCREASEING THE BLOOD FOLATE LEVELS AMONG WOMEN OF REPRODUCTIVE AGE – AN INTERVENTIONAL STUDY

Subject Name:

Date of Birth/Age:

Address of the Subject:

Qualification:

1. I Confirm that I have read and understood the Information sheet

2. I Understood that My participation in the study is Voluntarily

3. I agree to participation in the above study

Signature of the Patient/LAR

Date:

Signature of Investigator

Date:

<u>సమ్మతిపత్రం</u>

అధ్యయనశీర్షిక:

పునరుత్పత్తివయస్సుగలస్త్రీలలోరక్తపుఫోలేట్స్థాయిలనుపెంచడంలోఫోలిక్యా
సిడ్కలిపినసాల్ట్ (□□□□□□□□)యొక్కసమర్థత.

పేరు:

పుట్టినతేదీ/వయస్సు:

చిరునామా:

అర్హత:

1. నేనుసమాచారపత్రాన్నిచదివిఅర్థంచేసుకున్నాననిధృవీకరిస్తున్నాను

2. అధ్యయనంలోనాభాగస్వామ్యంస్వచ్ఛందంగాఅనినేనుఅర్థంచేసుకున్నాను

3. పైఅధ్యయనంలోపాల్గొనడానికినేనుఅంగీకరిస్తున్నాను

□□□□□□□□□□□□□□□□□□□□□□□□□□□□

తేదీతేదీ
