# Supplementary material for: Folic Acid–Fortified Iodized Salt and Serum Folate Levels in Reproductive-Aged Women of Rural India: A Nonrandomized Controlled Trial
Source: JAMA Netw Open. 2024 Mar 8;7(3):e241777. doi: 10.1001/jamanetworkopen.2024.1777 (PMC10924245; doi:10.1001/jamanetworkopen.2024.1777)
Supplement: Supplement 2. — Data Sharing Statement [file jamanetwopen-e241777-s002.pdf]

Data Sharing Statement

Pattisapu. Folic Acid–Fortified Iodized Salt and Serum Folate Levels in Reproductive-Aged Women of Rural India. *JAMA Netw Open*. Published March 08, 2024.
doi:10.1001/jamanetworkopen.2024.1777

Data
Data available: Yes
Data types: Deidentified participant data
How to access data: We will provide a link to the deidentified data set upon review of the request
When available: beginning date: 01-01-2024, end date: 12-31-2024

Supporting Documents
Document types: None

Additional Information
Who can access the data: To investigators who request the information upon review
Types of analyses: our laboratory data and patient characteristics
Mechanisms of data availability: with investigator support
